# Development and validation of a machine learning-based prediction model for anemia risk in patients with primary lung cancer receiving platinum-based chemotherapy: a multicenter study

**DOI:** 10.3389/fonc.2026.1948751

**Published:** 2026-09-30

**Authors:** Kaining Zhang, Xiao Li, Xiao Zhang, Xia Wang, Huijuan Zhang, Shuying Liu, Yating Gao, Jierong Yin, Xiaofei Li, Dongfeng Zhang

**Affiliations:** 1 Department of Oncology, Linfen Central Hospital, Linfen, Shanxi, China; 2 Department of Medical Records and Statistics, Affiliated Banan Hospital of Chongqing Medical University, Chongqing, China

**Keywords:** anemia, LightGBM, machine learning, primary lung cancer, SHAP

## Abstract

**Background:**

Anemia represents a frequent and clinically burdensome complication in patients with primary lung cancer receiving platinum-based regimens, yet robust risk stratification tools that integrate multidimensional clinical data and undergo external validation remain scarce. This multicenter study sought to develop and externally validate a machine learning-based predictive model for CIA in this population.

**Methods:**

A total of 3,434 patients with primary lung cancer were retrospectively enrolled from two geographically distinct institutions, with 3,097 assigned to the training/internal validation set (7:3 split) and 337 retained as an independent external validation set. Variables with missing rates exceeding 30% were excluded, and remaining missing data were imputed using multiple imputation by chained equations (MICE). Thirteen candidate predictors were preselected via univariate screening and least absolute shrinkage and selection operator (LASSO) regression. Nine machine learning algorithms were developed and comparatively evaluated using area under the receiver operating characteristic curve (AUC), calibration plots, and decision curve analysis. Model interpretability was achieved through SHapley Additive exPlanations (SHAP). The primary outcome was defined as hemoglobin levels < 100 g/L (CTCAE Grade ≥ 2) recorded during the first two cycles of chemotherapy.

**Results:**

Among the nine candidate models, LightGBM achieved the highest discriminative performance, with AUCs of 0.871 (95% CI: 0.852–0.891) in the training set, 0.797 (95% CI: 0.763–0.831) in internal validation, and 0.684 (95% CI: 0.626–0.742) in the external validation set derived from Linfen Central Hospital. Calibration and decision curve analyses consistently demonstrated superior agreement and net clinical benefit for LightGBM across all three datasets. SHAP-based interpretation identified lymphocyte count, D-dimer, alanine aminotransferase, total bilirubin, sex, and albumin as the six most influential predictors, with D-dimer, female sex, and outpatient admission positively associated with anemia risk, whereas lymphocyte count, liver function indices, and albumin showed inverse associations.

**Conclusions:**

The LightGBM-based model developed herein demonstrates acceptable and moderate predictive performance, satisfactory calibration, and potential clinical utility across geographically diverse populations, offering a preliminary risk-stratification tool for early identification of lung cancer patients at elevated anemia risk prior to chemotherapy initiation. However, given the modest external validation performance, cautious interpretation and further refinement are warranted before widespread clinical deployment.

## Introduction

Lung cancer remains the most frequently diagnosed cancer and the leading cause of cancer-related mortality worldwide, accounting for approximately 2.48 million new cases (12.4% of all cancers) and 1.82 million deaths (18.7% of all cancer deaths) in 2022 according to GLOBOCAN estimates (1, 2). In China alone, over 40% of global lung cancer cases and deaths occur (3). Although advances in targeted therapy and immunotherapy have improved outcomes for subsets of patients, platinum-based systemic chemotherapy continues to serve as the cornerstone of treatment for locally advanced and metastatic non-small cell lung cancer (NSCLC) (4, 5).

However, chemotherapy frequently induces a spectrum of hematological toxicities, among which anemia is one of the most prevalent and clinically significant (6). Data from the European Cancer Anaemia Survey (ECAS) showed that over 80% of lung cancer patients receiving platinum-based regimens developed anemia during treatment (7). A recent real-world study of 403 NSCLC patients across 1,659 chemotherapy cycles reported that adverse events occurred in more than half of systemic anticancer treatment episodes, with myelosuppression affecting 18.3% of cycles (8). Anemia not only compromises patients’ quality of life but may also necessitate chemotherapy dose reductions or treatment delays, thereby undermining antitumor efficacy (9). Therefore, early identification of high-risk patients for chemotherapy-induced anemia is of critical clinical importance.

Although several prediction models for chemotherapy-related adverse events in lung cancer have been developed, notable limitations persist. Conventional logistic regression-based approaches predominate in existing tools, yet they exhibit limited capacity to capture complex non-linear interactions inherent in multidimensional clinical data (10). Machine learning (ML) models have demonstrated improved predictive performance in lung cancer patients receiving chemotherapy. For instance, Sun et al. developed a risk prediction model for lung infection after chemotherapy using multiple ML algorithms and found that the random forest (RF) model achieved superior performance with an area under the receiver operating characteristic curve (AUC) of 0.897 (95% CI: 0.875–0.914) and an F1 score of 0.795 (11). Despite these encouraging results, most models—including this one—were developed using single-center data and lacked rigorous external validation. Furthermore, existing models predominantly incorporate limited feature sets, with insufficient integration of coagulation, inflammatory, and nutritional markers (12). Although ML approaches have been applied to toxicity prediction in patients with locally advanced NSCLC receiving definitive chemoradiation, with extreme gradient boosting (XGBoost) achieving an AUC of 0.739 for pulmonary toxicity and RF achieving an AUC of 0.706 for cardiac toxicity, their focus has not specifically addressed chemotherapy-induced anemia (13). Collectively, a dedicated chemotherapy-induced anemia prediction model that integrates comprehensive multidimensional variables and has undergone rigorous external validation remains conspicuously absent (14).

Given the aforementioned limitations, the development of a robust, externally validated prediction model specifically for chemotherapy-induced anemia in lung cancer patients receiving chemotherapy is urgently warranted. Therefore, the present study aims to construct and validate a machine learning-based predictive model for anemia risk in this population, integrating multidimensional clinical variables to improve risk stratification. The ultimate goal is to provide clinicians with a practical tool for early identification of high-risk patients, thereby enabling timely preventive and therapeutic interventions.

## Methods

### Study design and study population

The present retrospective investigation leveraged electronic medical record (EMR) from two geographically distinct institutions—the Affiliated Banan Hospital of Chongqing Medical University and Linfen Central Hospital—to construct a study population of patients hospitalized for lung cancer. Data accrual occurred across non-overlapping temporal windows: from January 2020 through December 2025 at the former site, and from January 2018 through December 2025 at the latter. In instances of repeat admissions, only the index hospitalization was considered for inclusion. Eligibility was determined by prespecified criteria, namely: (i) attained age of 18 years or older; and (ii) a definitive diagnosis of primary lung cancer necessitating hospital-based care. Patients were rendered ineligible if they met any of the following exclusionary conditions: (i) absence of chemotherapy administration; (ii) coexisting hematological comorbidities, such as thalassemia, aplastic anemia, or myelodysplastic syndrome; or (iii) severe compromise of hepatic or renal function. Application of these selection parameters yielded a final analytical cohort of 3,434 patients across the two participating centers. Due to the retrospective nature of the electronic medical record data, we were unable to definitively distinguish between pre-existing anemia and anemia that developed after chemotherapy initiation. The Banan subsample (n = 3,097) underwent randomized allocation in a 7:3 split, generating a training set of 2,167 patients and an internal validation set of 930 patients. Concurrently, the Linfen subsample (n = 337) was reserved as a fully independent, geographically external validation set. The comprehensive schematic of the sequential enrollment and exclusion workflow is illustrated in **Figure 1**.

**Figure 1:**
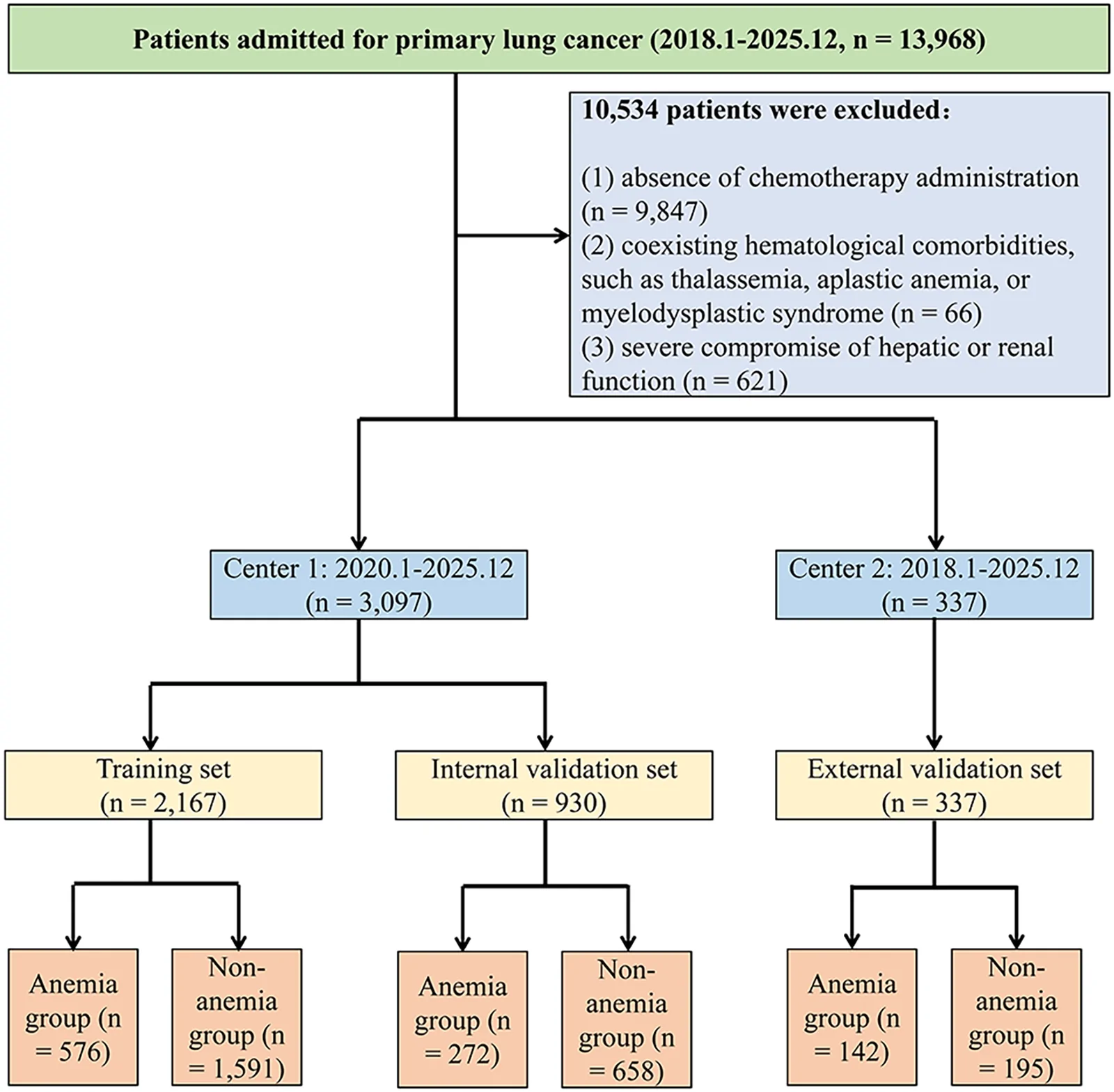
Flowchart of the study design. The diagram illustrates the sequential enrollment and exclusion workflow across the two centers, the randomized split into training and internal validation sets, and the retention of the independent external validation set. Regarding the chronological sequence of the study: all baseline clinical and laboratory predictors were collected prior to the initiation of the first cycle of platinum-based chemotherapy. The primary outcome (anemia) was assessed during the first two cycles of chemotherapy following treatment initiation.

Ethical clearance for the study protocol was secured from the respective review boards of both institutions, namely the Affiliated Banan Hospital of Chongqing Medical University (approval reference: BNLL-KY-2026-027) and Linfen Central Hospital (approval reference: LY2026-35-01). The investigation was carried out in strict conformity with the principles articulated in the Declaration of Helsinki (15). In light of the observational retrospective design and the exclusive utilization of anonymized data for all analytical procedures, the requirement for patient-informed consent was formally exempted.

### Outcome definition

The primary outcome was the presence of anemia, defined according to the Common Terminology Criteria for Adverse Events (CTCAE) version 5.0 as a hemoglobin level below 100 g/L (Grade 2 or higher), recorded during the first two cycles of platinum-based chemotherapy (16). Given the limitations of the retrospective EMR dataset, we were unable to definitively determine whether anemia in all cases strictly occurred after the initiation of chemotherapy or was pre-existing at the time of the index admission. We therefore acknowledge that the outcome represents the overall anemia burden during early chemotherapy, rather than strictly incident chemotherapy-induced anemia. Consequently, our model is intended as a risk stratification tool for identifying patients at elevated anemia risk prior to treatment, rather than a tool for predicting the exact hematological toxicity of a specific treatment protocol. Moreover, baseline hemoglobin levels were not consistently recorded in our structured EMR system across the participating centers. Consequently, we were unable to calculate the prevalence of baseline anemia or exclude patients with pre-existing anemia. Given that the primary outcome was assessed during the first two cycles of chemotherapy, the follow-up cutoff for outcome capture was defined as the completion date of the second chemotherapy cycle for each individual patient. Data for the final cohort were collected through December 2025.

### Data collection

Baseline clinical data for all study participants were retrieved from the EMR repositories of the two participating institutions. A comprehensive set of variables was extracted, encompassing the following domains: demographic attributes (age and sex), admission pathway, behavioral risk factors (smoking history and drinking history), comorbid conditions (surgical history, diabetes mellitus (DM), hypertension, coronary heart disease (CHD), chronic gastritis, and pneumonia), as well as an array of laboratory determinations. The latter comprised coagulation parameters (fibrinogen and D-dimer), hematological indices (platelet (PLT) count, mean platelet volume (MPV), platelet distribution width (PDW), neutrophil (NEUT) count, lymphocyte (LYM) count, and monocyte (MONO) count), renal function markers (blood urea nitrogen (BUN), creatinine (CREA), and uric acid (UA)), hepatic function panels (alanine aminotransferase (ALT), aspartate aminotransferase (AST), alkaline phosphatase (ALP), total bilirubin (TBIL), albumin (ALB), and globulin (GLB)), and serum electrolytes (potassium (K), phosphorus (P), chloride (Cl), sodium (Na), and calcium (Ca)). All laboratory measurements were derived from fasting venous blood specimens drawn prior to the initiation of the first cycle of platinum-based chemotherapy at the time of the index admission and were processed following standardized protocols at the respective clinical laboratories of each center. To ensure the temporality of our study design and avoid potential reverse causality, no laboratory variables were collected after the administration of chemotherapy.

### Data imputation and feature selection

To optimize the utilization of available data, variables with a missing proportion exceeding 30% were excluded before the imputation procedure. For the remaining variables, missing entries were handled via multiple imputation by chained equations (MICE), an approach that preserves the distributional characteristics of the original data more faithfully than alternative imputation techniques (17). The detailed list of all variables, along with their respective missing rates, is provided in **Supplementary Table 1**. The threshold of 30% was chosen based on established methodological guidance, which recommends that when missing proportions exceed 20-40%, multiple imputation combined with sensitivity analysis is mandatory, while proportions beyond 40% warrant considering variable exclusion (17, 18).

Feature selection represents a pivotal stage in the model development workflow, conferring multiple benefits including enhanced predictive performance, reduced computational burden, improved generalizability, and augmented model interpretability. The selection process was initiated with univariate analyses conducted on the training set to identify candidate predictors exhibiting significant associations with the outcome of interest. Variables that reached statistical significance in this univariate screening were subsequently subjected to least absolute shrinkage and selection operator (LASSO) regression for further dimensionality reduction. The optimal tuning parameter λ was selected via ten-fold cross-validation, applying the one-standard-error rule (1-SE rule) to identify the most parsimonious model within one standard error of the minimum cross-validated error.

### Model development and evaluation

A total of nine mainstream ML algorithms were implemented for model development, comprising light gradient boosting machine (LightGBM), XGBoost, gradient boosting decision tree (GBDT), RF, logistic regression (LR), decision tree (DT), K-nearest neighbors (KNN), artificial neural network (ANN), and support vector machine (SVM). All candidate models underwent performance assessment on both the training and validation sets. To facilitate cross-model comparisons, a comprehensive panel of performance metrics was adopted, including the AUC with its accompanying 95% confidence interval (CI), sensitivity, specificity, F1 score, and Youden’s index, with AUC designated as the primary measure of discriminative capacity. Calibration plots were generated to evaluate the concordance between model-estimated probabilities and the empirically observed outcome rates. In parallel, decision curve analysis (DCA) was conducted to quantify the net clinical benefit accruing from each model across a spectrum of clinically relevant threshold probabilities, thereby capturing the trade-off between expected gains and potential harms associated with prediction-guided decisions.

To ensure a rigorous and objective selection of the final model, we adopted a comprehensive multi-criteria decision-making framework. Although AUC served as the primary metric for initial screening, the final model was not selected solely based on maximizing AUC in a single dataset. Instead, we systematically evaluated all nine algorithms across three key dimensions (1): Discrimination (AUC and 95% CI) (2); Calibration (visual inspection of calibration plots and Brier scores); and (3) Clinical Utility (net benefit derived from DCA). The final model was selected based on its overall balanced performance across these dimensions in both internal and external validation sets, rather than relying solely on a single metric in one dataset. For interpretability of the best-performing model, Shapley additive explanations (SHAP) were applied (19, 20). Grounded in cooperative game theory, SHAP quantifies the directional contribution and relative importance of each predictor to the model’s output. The resultant SHAP values enable the identification of dominant features that materially drive model predictions. To further elucidate the effect of individual covariates, SHAP force plots were generated for representative cases, visually depicting the marginal contribution of each variable to the predicted outcome.

### Statistical analysis

All statistical computations were carried out using R (version 4.3.3) and Python (version 3.12.3). Normality of continuous variables was assessed prior to group comparisons: variables conforming to a normal distribution were summarized as mean ± standard deviation (SD) and compared across groups via Student’s t-test, whereas those deviating from normality were reported as median with interquartile range (IQR) and evaluated using the Mann-Whitney U test. Categorical variables were expressed as frequencies with corresponding percentages and examined for between-group differences using the chi-square test. A two-tailed *P* value < 0.05 was defined as the threshold for statistical significance.

To further evaluate the potential for multicollinearity among the predictors, variance inflation factor (VIF) diagnostics were applied to the full set of candidate variables prior to variable selection (i.e., before LASSO regression) to assess the degree of multicollinearity among the original variables. A VIF value below 5 for all included variables was considered indicative of no substantial multicollinearity. In parallel, a correlation heatmap was generated, and correlation coefficients were computed for continuous variables to visually depict pairwise associations. Additionally, restricted cubic spline (RCS) regression was performed to examine the presence of non-linear relationships between continuous predictors and the outcome (21).

## Results

### Characteristics of the enrolled patients

Between January 2018 and December 2025, a total of 3,434 eligible participants were enrolled across the two participating centers. In the combined training (n = 2,167) and internal validation (n = 930) sets, the median age was 64 years (IQR: 57–69 years), with males accounting for 2,117 (68.36%) and females for 980 (31.64%) of the pooled sample (**Table 1**). Outpatient referral constituted the admission pathway for 53.21% of the study population. The proportions of patients reporting smoking history, drinking history, and surgical history were 48.11%, 19.57%, and 22.89%, respectively. With respect to comorbid conditions, the three most frequently observed diagnoses were hypertension (22.51%), DM (13.30%), and pneumonia (11.33%). The results of multiple imputation for variables in the training and internal validation sets are detailed in **Supplementary Tables 2** and **3**, respectively. For the vast majority of variables, no statistically significant discrepancies were detected between pre- and post-imputation distributions (all *P* > 0.05), suggesting that the imputation procedure performed satisfactorily.

**Table 1:** Baseline characteristics of the training and internal validation sets.

Variables	Total (n = 3,097)	Training set (n = 2,167)	Internal validation set (n = 930)	*P* value
Age (years)	64 (57, 69)	63 (56, 69)	64 (57, 69)	0.265
Sex				0.722
male	2117 (68.36)	1486 (68.57)	631 (67.85)	
female	980 (31.64)	681 (31.43)	299 (32.15)	
Admission pathway				0.285
emergency	1449 (46.79)	1028 (47.44)	421 (45.27)	
outpatient	1648 (53.21)	1139 (52.56)	509 (54.73)	
Smoking history				0.810
no	1607 (51.89)	1128 (52.05)	479 (51.51)	
yes	1490 (48.11)	1039 (47.95)	451 (48.49)	
Drinking history				0.218
no	2491 (80.43)	1730 (79.83)	761 (81.83)	
yes	606 (19.57)	437 (20.17)	169 (18.17)	
Surgical history				0.809
no	2388 (77.11)	1674 (77.25)	714 (76.77)	
yes	709 (22.89)	493 (22.75)	216 (23.23)	
DM				0.006
no	2685 (86.7)	1903 (87.82)	782 (84.09)	
yes	412 (13.3)	264 (12.18)	148 (15.91)	
Hypertension				0.072
no	2400 (77.49)	1699 (78.4)	701 (75.38)	
yes	697 (22.51)	468 (21.6)	229 (24.62)	
CHD				0.339
no	2940 (94.93)	2063 (95.2)	877 (94.3)	
yes	157 (5.07)	104 (4.8)	53 (5.7)	
Chronic gastritis				0.472
no	2865 (92.51)	2010 (92.75)	855 (91.94)	
yes	232 (7.49)	157 (7.25)	75 (8.06)	
Pneumonia				0.111
no	2746 (88.67)	1908 (88.05)	838 (90.11)	
yes	351 (11.33)	259 (11.95)	92 (9.89)	
Fibrinogen (g/L)	3.94 (3.28, 4.85)	3.9 (3.27, 4.8)	4.01 (3.3, 4.98)	0.047
D-dimer (mg/L)	0.45 (0.31, 0.8)	0.44 (0.31, 0.8)	0.48 (0.32, 0.8)	0.060
PLT count (×10^9^/L)	211 (161, 275)	213 (162, 272.5)	208 (155, 280)	0.537
MPV (fL)	9.2 (8.5, 10.1)	9.2 (8.5, 10.1)	9.2 (8.5, 10)	0.499
PDW (%)	16.1 (15.7, 16.4)	16.1 (15.7, 16.4)	16.1 (15.7, 16.4)	0.296
NEUT count (×10^9^/L)	3.44 (2.38, 4.72)	3.47 (2.39, 4.72)	3.41 (2.35, 4.74)	0.319
LYM count (×10^9^/L)	1.06 (0.76, 1.42)	1.06 (0.77, 1.42)	1.05 (0.75, 1.4)	0.455
MONO count (×10^9^/L)	0.42 (0.31, 0.55)	0.42 (0.32, 0.55)	0.42 (0.31, 0.55)	0.959
BUN (mmol/L)	2.38 (1.96, 2.88)	2.38 (1.96, 2.88)	2.38 (1.92, 2.88)	0.706
CREA (μmol/L)	62 (52, 73)	62 (52, 73)	62 (53, 73)	0.842
UA (μmol/L)	324 (270, 384)	324 (272, 385)	322 (266.25, 381)	0.375
ALT (U/L)	20 (14, 28)	20 (14, 28)	19 (14, 27)	0.233
AST (U/L)	22 (18, 28)	23 (18, 28)	22 (18, 28)	0.196
ALP (U/L)	80 (66, 97)	81 (66, 97.5)	80 (65, 96)	0.516
TBIL (μmol/L)	8.9 (7, 11.8)	9 (7, 11.9)	8.8 (7, 11.6)	0.290
ALB (g/L)	41.04 (38.65, 43.32)	41.08 (38.71, 43.43)	40.85 (38.56, 43.08)	0.061
GLB (g/L)	27.29 (24.8, 30.09)	27.3 (24.78, 30.09)	27.27 (24.87, 30.06)	0.802
K (mmol/L)	4.06 (3.81, 4.28)	4.06 (3.82, 4.28)	4.04 (3.8, 4.28)	0.243
P (mmol/L)	1.15 (1.01, 1.29)	1.14 (1.01, 1.28)	1.16 (1.01, 1.31)	0.127
Cl (mmol/L)	105 (103, 107)	105 (103, 107)	105 (103, 107)	0.701
Na (mmol/L)	140 (138, 141)	140 (138, 141)	139.8 (138, 141)	0.658
Ca (mmol/L)	2.26 (2.18, 2.34)	2.26 (2.18, 2.34)	2.25 (2.17, 2.33)	0.045

DM, diabetes mellitus; CHD, coronary heart disease; PLT, platelet; MPV, mean platelet volume; PDW, platelet distribution width; NEUT, neutrophil; LYM, lymphocyte; MONO, monocyte; BUN, blood urea nitrogen; CREA, creatinine; UA, uric acid; ALT, alanine aminotransferase; AST, aspartate aminotransferase; ALP, alkaline phosphatase; TBIL, total bilirubin; ALB, albumin; GLB, globulin; K, potassium; P, phosphorus; Cl, chloride; Na, sodium; Ca, calcium.

### Feature selection

Within the training set, patients were categorized into two groups: those with anemia (n = 576, 26.58%) and those without (n = 1,591, 73.42%). The median age was 62 years (IQR: 55–69) for the anemia group and 64 years (IQR: 57–70) for the non-anemia group. A comprehensive comparison of all baseline variables between the two groups is summarized in **Table 2**. Relative to the non-anemia group, anemic patients tended to be younger (*P* = 0.01) and exhibited a significantly higher female proportion (43.92% vs. 26.90%, *P* < 0.001). Additionally, the two groups differed significantly with respect to smoking history, hypertension, fibrinogen, D-dimer, PLT count, MPV, PDW, NEUT count, LYM count, BUN, CREA, UA, ALT, AST, TBIL, ALB, P, Na, and Ca (all *P* < 0.05).

**Table 2:** Comparison of variables between anemia and non-anemia groups in the training set.

Variables	Total (n = 2,167)	Anemia group (n = 576)	non-Anemia group (n = 1,591)	*P* value
Age (years)	63 (56, 69)	62 (55, 69)	64 (57, 70)	0.01
Sex				< 0.001
male	1486 (68.57)	323 (56.08)	1163 (73.1)	
female	681 (31.43)	253 (43.92)	428 (26.9)	
Admission pathway				0.001
emergency	1028 (47.44)	240 (41.67)	788 (49.53)	
outpatient	1139 (52.56)	336 (58.33)	803 (50.47)	
Smoking history				< 0.001
no	1128 (52.05)	341 (59.2)	787 (49.47)	
yes	1039 (47.95)	235 (40.8)	804 (50.53)	
Drinking history				0.125
no	1730 (79.83)	473 (82.12)	1257 (79.01)	
yes	437 (20.17)	103 (17.88)	334 (20.99)	
Surgical history				0.184
no	1674 (77.25)	433 (75.17)	1241 (78)	
yes	493 (22.75)	143 (24.83)	350 (22)	
DM				0.276
no	1903 (87.82)	498 (86.46)	1405 (88.31)	
yes	264 (12.18)	78 (13.54)	186 (11.69)	
Hypertension				0.010
no	1699 (78.4)	474 (82.29)	1225 (77)	
yes	468 (21.6)	102 (17.71)	366 (23)	
CHD				0.516
no	2063 (95.2)	545 (94.62)	1518 (95.41)	
yes	104 (4.8)	31 (5.38)	73 (4.59)	
Chronic gastritis				0.480
no	2010 (92.75)	530 (92.01)	1480 (93.02)	
yes	157 (7.25)	46 (7.99)	111 (6.98)	
Pneumonia				0.194
no	1908 (88.05)	498 (86.46)	1410 (88.62)	
yes	259 (11.95)	78 (13.54)	181 (11.38)	
Fibrinogen (g/L)	3.9 (3.27, 4.8)	4.14 (3.48, 5.07)	3.79 (3.19, 4.71)	< 0.001
D-dimer (mg/L)	0.44 (0.31, 0.8)	0.61 (0.38, 1.12)	0.41 (0.3, 0.66)	< 0.001
PLT count (×10^9^/L)	213 (162, 272.5)	221.5 (162.75, 301)	209 (162, 268)	0.002
MPV (fL)	9.2 (8.5, 10.1)	9 (8.3, 9.7)	9.3 (8.6, 10.2)	< 0.001
PDW (%)	16.1 (15.7, 16.4)	16 (15.7, 16.3)	16.1 (15.8, 16.4)	0.006
NEUT count (×10^9^/L)	3.47 (2.39, 4.72)	3.17 (2.08, 4.51)	3.58 (2.51, 4.81)	< 0.001
LYM count (×10^9^/L)	1.06 (0.77, 1.42)	0.88 (0.67, 1.2)	1.12 (0.83, 1.49)	< 0.001
MONO count (×10^9^/L)	0.42 (0.32, 0.55)	0.4 (0.3, 0.56)	0.42 (0.32, 0.55)	0.185
BUN (mmol/L)	2.38 (1.96, 2.88)	2.25 (1.88, 2.83)	2.38 (1.96, 2.92)	0.01
CREA (μmol/L)	62 (52, 73)	59 (48, 70)	63 (54, 73)	< 0.001
UA (μmol/L)	324 (272, 385)	300 (245, 362.25)	332 (283, 391)	< 0.001
ALT (U/L)	20 (14, 28)	17 (13, 24)	21 (15, 30)	< 0.001
AST (U/L)	23 (18, 28)	21 (17, 27)	23 (19, 29)	< 0.001
ALP (U/L)	81 (66, 97.5)	80 (65, 98)	81 (67, 97)	0.645
TBIL (μmol/L)	9 (7, 11.9)	7.95 (6.4, 10.4)	9.4 (7.3, 12.2)	< 0.001
ALB (g/L)	41.08 (38.71, 43.43)	40.1 (37.18, 42.5)	41.37 (39.24, 43.7)	< 0.001
GLB (g/L)	27.3 (24.78, 30.09)	27.26 (24.92, 30.39)	27.3 (24.72, 29.96)	0.282
K (mmol/L)	4.06 (3.82, 4.28)	4.06 (3.8, 4.3)	4.06 (3.82, 4.27)	0.652
P (mmol/L)	1.14 (1.01, 1.28)	1.18 (1.04, 1.32)	1.13 (1, 1.27)	< 0.001
Cl (mmol/L)	105 (103, 107)	105 (102, 107)	105 (103, 107)	0.158
Na (mmol/L)	140 (138, 141)	139.3 (137.08, 141)	140 (138, 141)	0.015
Ca (mmol/L)	2.26 (2.18, 2.34)	2.24 (2.16, 2.33)	2.27 (2.2, 2.35)	< 0.001

DM, diabetes mellitus; CHD, coronary heart disease; PLT, platelet; MPV, mean platelet volume; PDW, platelet distribution width; NEUT, neutrophil; LYM, lymphocyte; MONO, monocyte; BUN, blood urea nitrogen; CREA, creatinine; UA, uric acid; ALT, alanine aminotransferase; AST, aspartate aminotransferase; ALP, alkaline phosphatase; TBIL, total bilirubin; ALB, albumin; GLB, globulin; K, potassium; P, phosphorus; Cl, chloride; Na, sodium; Ca, calcium.

The variables reaching statistical significance in the above between-group comparisons were then entered into LASSO regression for feature dimensionality reduction. Under ten-fold cross-validation with the one-standard-error rule, the optimal tuning parameter λ (lambda.1se = 0.01795907) was identified, which yielded a parsimonious set of 13 predictors with non-zero regression coefficients: age, sex, admission pathway, fibrinogen, D-dimer, MPV, NEUT count, LYM count, UA, ALT, TBIL, ALB, and P (**Figure 2**).

**Figure 2:**
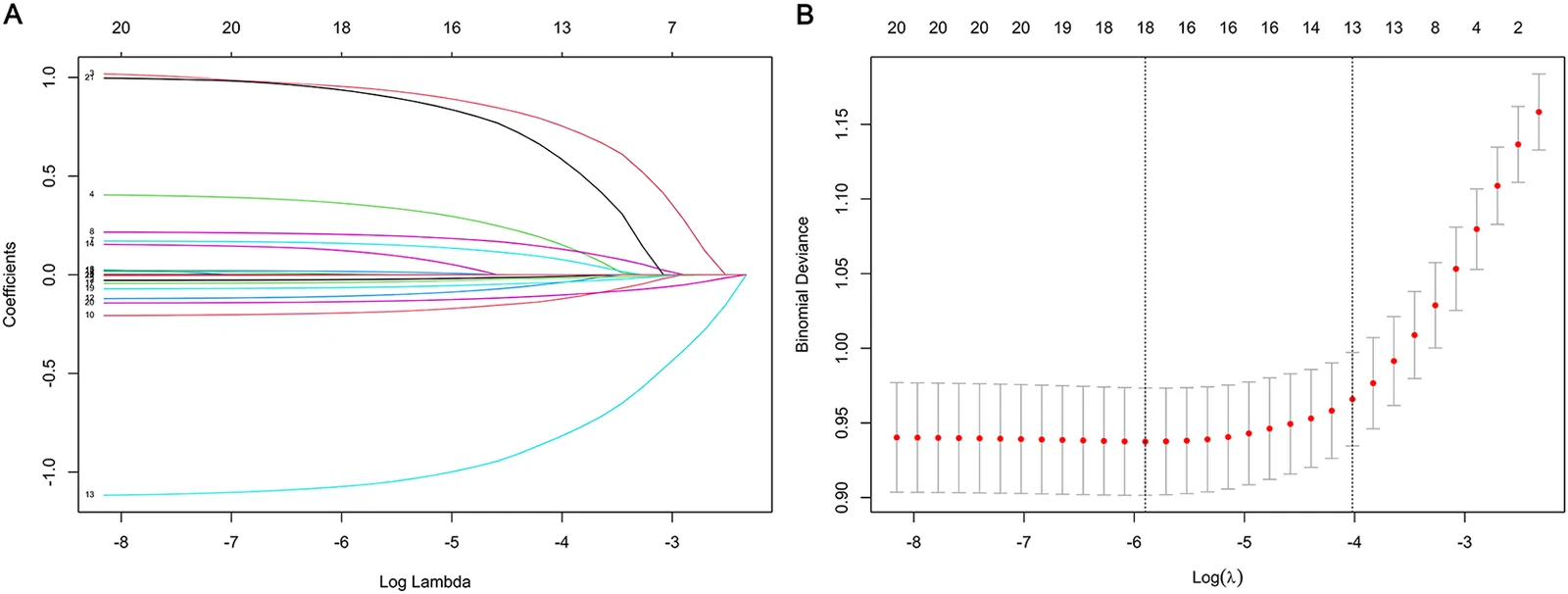
LASSO regression for feature selection. **(A)** Coefficient shrinkage paths for the 13 selected predictors, with each curve illustrating the trajectory of a variable’s coefficient as λ increases. **(B)** Cross-validation error plotted against log(λ), with vertical dashed lines indicating the λ value that minimizes the error (left) and the λ value within one standard error of the minimum (right, lambda.1se = 0.01795907).

To rigorously assess the potential for multicollinearity among the 13 retained predictors, we conducted both correlation analysis and collinearity diagnostics. As depicted in **Figure 3**, the absolute values of all pairwise correlation coefficients among continuous variables were below 0.35. The strongest correlation was observed between fibrinogen and ALB (r = −0.31), followed by age with ALB (r = −0.29) and ALB with D-dimer (r = −0.29); all other pairwise associations were weak to negligible. Furthermore, as shown in **Supplementary Table 4**, the VIF diagnostics conducted on the full set of initial candidate variables (pre-LASSO) revealed no substantial multicollinearity, with all VIF values remaining well below the conventional threshold of 5. Notably, relatively higher collinearity was observed between electrolyte parameters, specifically Na and Cl (VIF = 3.443 and 3.346, respectively). Despite this, the subsequent LASSO regression with ten-fold cross-validation and the one-standard-error rule automatically handled these correlated variables by penalizing their coefficients and retaining only the most informative predictors (e.g., P was retained while Na and Cl were excluded), thereby effectively mitigating the impact of multicollinearity. Taken together, these results confirm the absence of significant multicollinearity among the final set of predictors.

**Figure 3:**
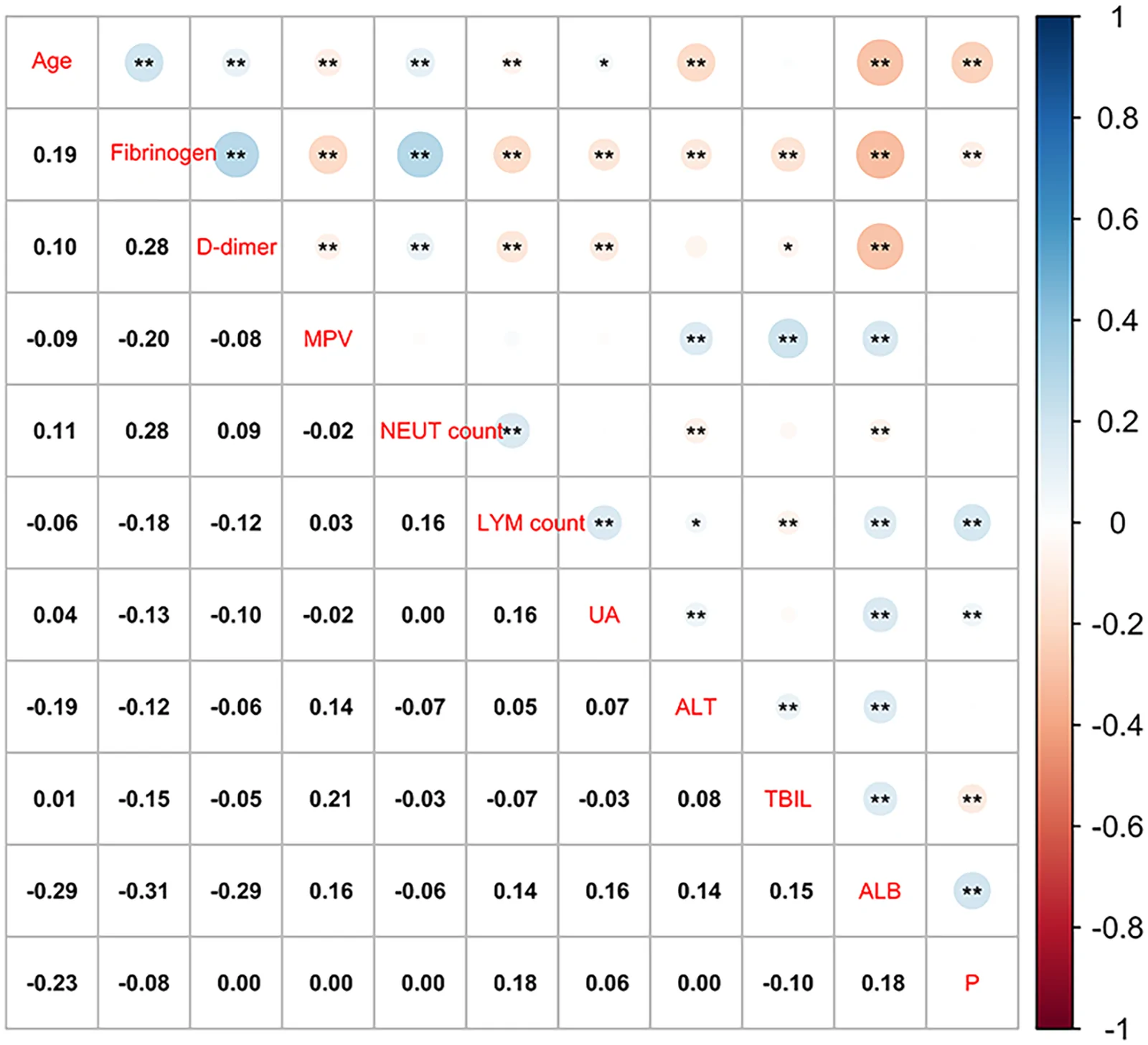
Heatmap depicting pairwise correlations among continuous predictors selected via LASSO. *: P < 0.05; **: P < 0.01.

### RCS analysis of continuous predictors

RCS regression, adjusted for potential confounders, was applied to the 11 continuous predictors retained by LASSO to elucidate their dose-response relationships with anemia risk (**Figure 4**). Regarding non-linearity, five variables exhibited significant departures from linearity: fibrinogen (*P*-nonlinear = 0.006), D-dimer (*P*-nonlinear = 0.001), NEUT count (*P*-nonlinear = 0.014), UA (*P*-nonlinear = 0.001), and TBIL (*P*-nonlinear < 0.001). Fibrinogen displayed a complex fluctuating pattern, with an initial increase peaking around 3.8 g/L, followed by a decline to a trough at approximately 4.8 g/L, and a subsequent gradual rise at higher concentrations; D-dimer demonstrated a progressively increasing risk with rising concentrations, though the upward trajectory gradually attenuated at higher levels; NEUT count exhibited a decreasing trend that leveled off beyond approximately 6.5 × 10^9^/L, with no further decline at higher counts; UA exhibited a U-shaped pattern with elevated risk at both low and high levels; and TBIL demonstrated a sharp initial decline followed by a plateau. By contrast, age (*P*-nonlinear = 0.167), MPV (*P*-nonlinear = 0.788), LYM count (*P*-nonlinear = 0.959), ALT (*P*-nonlinear = 0.267), ALB (*P*-nonlinear = 0.054), and P (*P*-nonlinear = 0.648) did not reach significance for non-linearity, indicating predominantly linear associations with anemia risk over the observed ranges.

**Figure 4:**
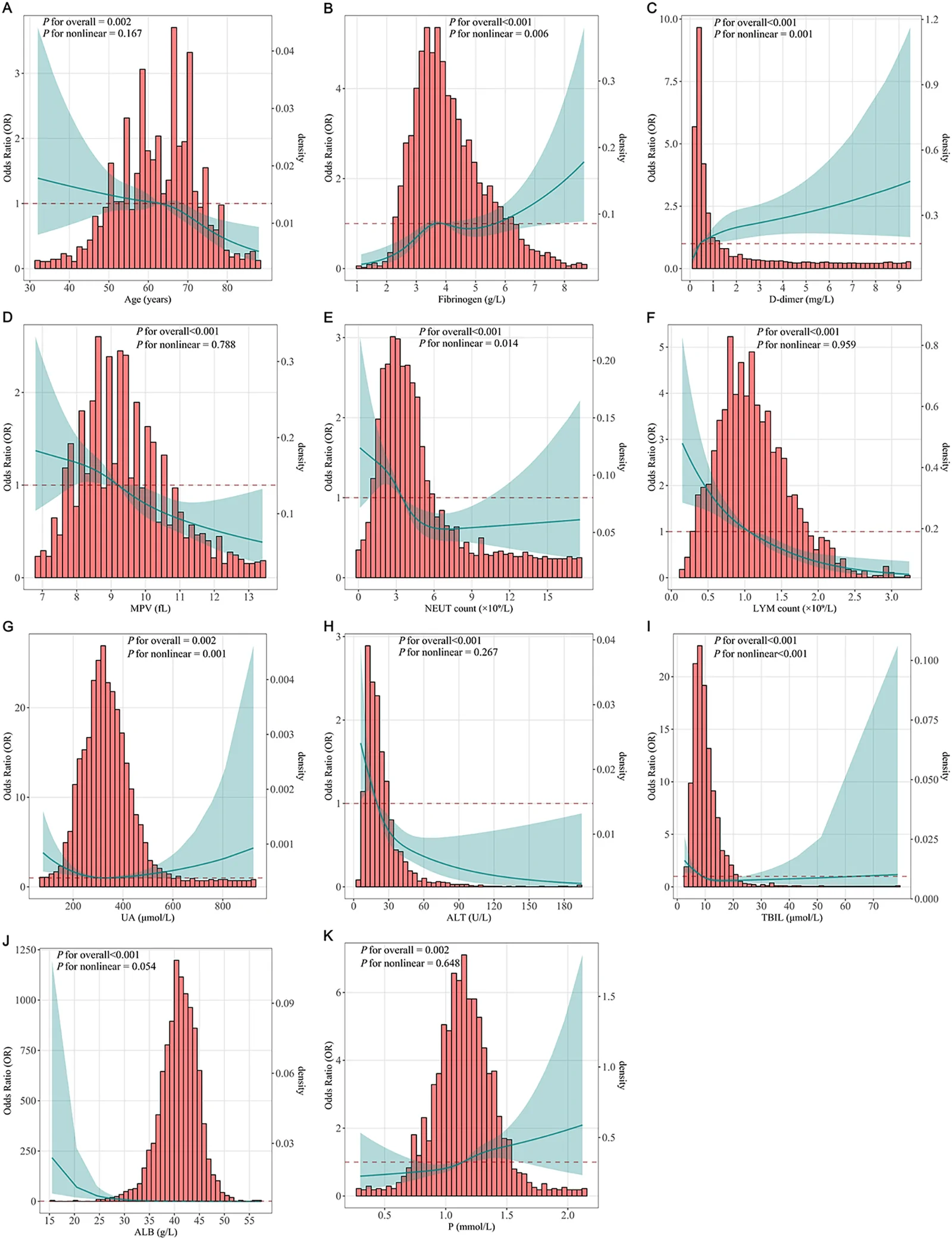
RCS analysis for dose-response relationships. **(A)** Age; **(B)** Fibrinogen; **(C)** D-dimer; **(D)** MPV; **(E)** NEUT count; **(F)** LYM count; **(G)** UA; **(H)** ALT; **(I)** TBIL; **(J)** ALB; **(K)** P.

### Model performance and evaluation

Nine ML algorithms were developed and rigorously benchmarked across training, internal validation, and external validation sets. Performance metrics for each model in the training set are compiled in **Table 3**. Among the candidates, LightGBM emerged as the top-performing classifier, attaining a training-set AUC of 0.871 (95% CI: 0.852–0.891), with corresponding sensitivity, specificity, Youden index, and F1 score of 0.863, 0.723, 0.586, and 0.657, respectively. In the internal and external validation sets, LightGBM yielded AUCs of 0.797 (95% CI: 0.763–0.831) and 0.684 (95% CI: 0.626–0.742), with full performance breakdowns available in **Supplementary Tables 5** and **6**; notably, while LightGBM demonstrated the highest discriminative ability in the training set, its performance in the internal validation set was numerically comparable to several competing models (e.g., LR, ANN, SVM), and it exhibited a moderate degree of discrimination in the external validation set. Receiver operating characteristic curves for all nine models across the three datasets are depicted in **Figures 5A–C**, while the corresponding calibration plots (**Figures 6A–C**) reveal that LightGBM consistently aligns most closely with the ideal 45° diagonal, reflecting superior calibration relative to its counterparts. DCA (**Figures 7A–C**) further establish that LightGBM affords the highest net benefit over a clinically relevant spectrum of threshold probabilities in all three datasets, thereby supporting its potential value as a preliminary risk-stratification tool, though with moderate discriminative accuracy in external settings.

**Table 3:** Performance metrics of nine ML models in the training set.

Model	AUC (95%CI)	Sensitivity	Specificity	Youden index	F1 Score
LightGBM	0.871 (0.852-0.891)	0.863	0.723	0.586	0.657
XGBoost	0.864 (0.844-0.884)	0.847	0.725	0.573	0.65
GBDT	0.849 (0.829-0.870)	0.762	0.786	0.548	0.648
RF	0.813 (0.790-0.835)	0.755	0.697	0.452	0.583
LR	0.800 (0.777-0.824)	0.745	0.725	0.47	0.595
DT	0.769 (0.745-0.793)	0.674	0.728	0.402	0.556
KNN	0.815 (0.792-0.837)	0.698	0.769	0.467	0.597
ANN	0.801 (0.778-0.825)	0.717	0.737	0.454	0.587
SVM	0.831 (0.809-0.853)	0.825	0.686	0.51	0.613

AUC, area under the ROC curve; CI, confidence interval; LightGBM, light gradient boosting machine; XGBoost, extreme Gradient Boosting; GBDT, gradient boosting decision tree; RF, random forest; LR, logistic regression; DT, decision tree; KNN, K-nearest neighbors; ANN, artificial neural network; SVM, support vector machine.

**Figure 5:**
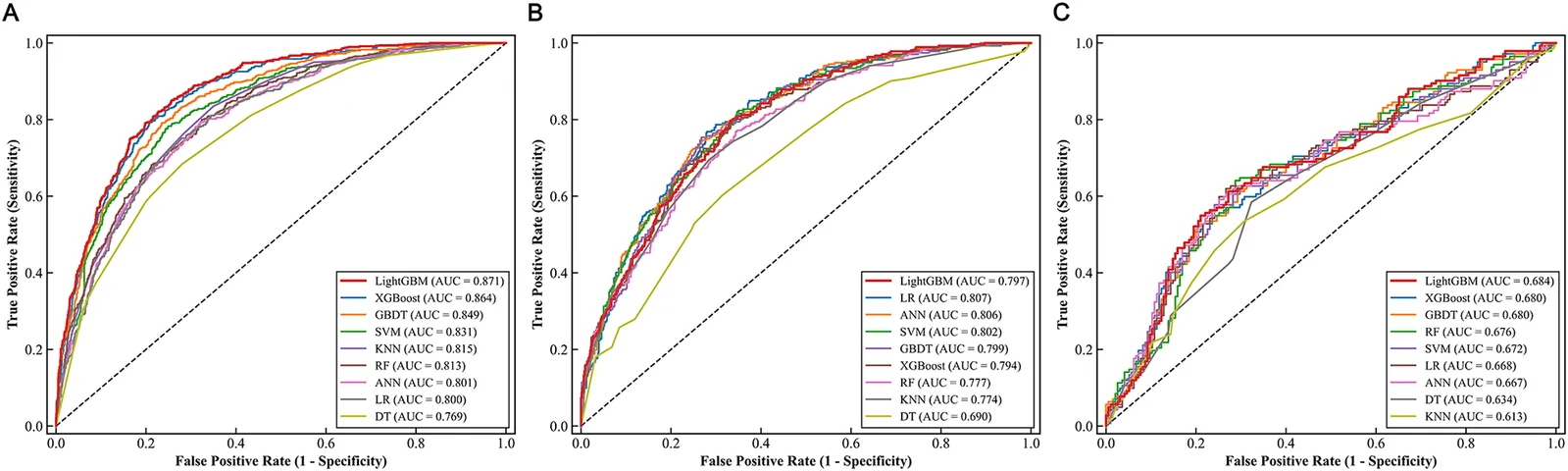
ROC curves of nine ML models in the training and validation sets. **(A)** Training set; **(B)** Internal validation set; **(C)** External validation set.

**Figure 6:**
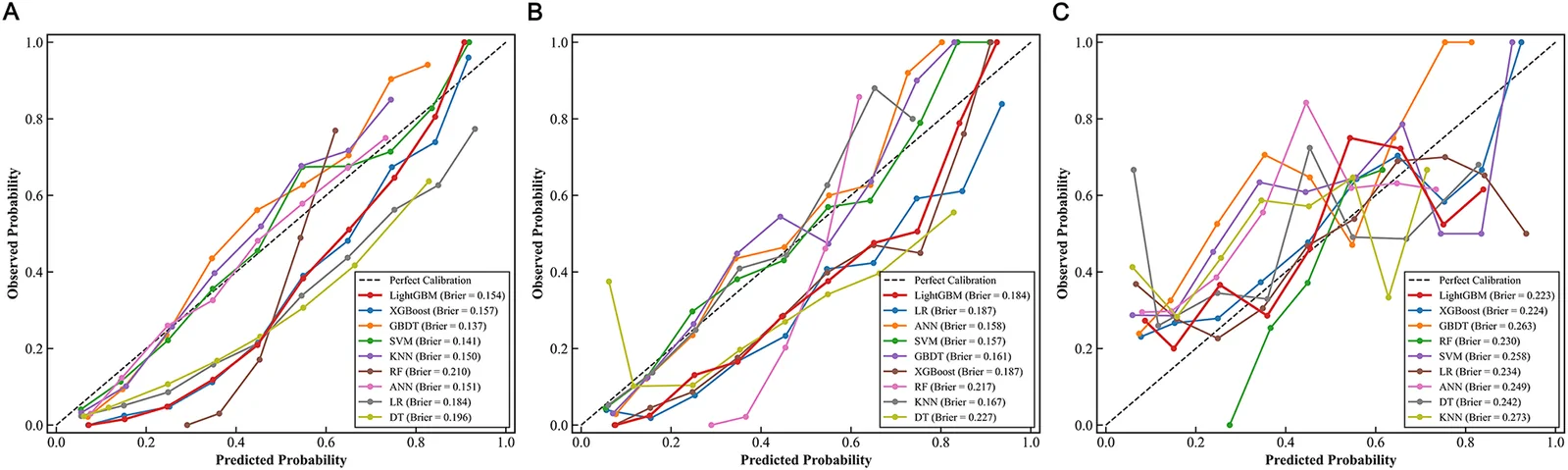
Calibration curves of nine ML models in the training and validation sets. **(A)** Training set; **(B)** Internal validation set; **(C)** External validation set.

**Figure 7:**
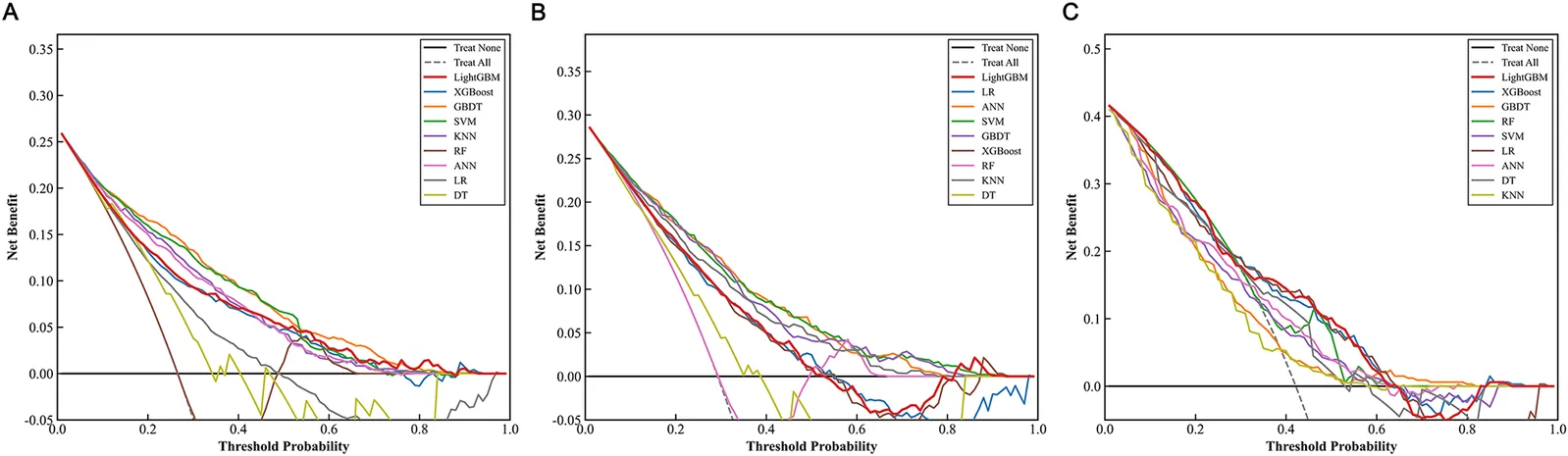
DCA of nine ML models in the training and validation sets. **(A)** Training set; **(B)** Internal validation set; **(C)** External validation set.

### Comparison of baseline characteristics between validation sets

To investigate the reasons for the performance decline in the external validation set, we systematically compared baseline characteristics between the internal validation set (n = 930) and the external validation set (n = 337). The results are presented in **Supplementary Table 7**. Notably, our analysis revealed substantial heterogeneity between the two independent sets. The external validation set exhibited a significantly higher proportion of male patients (standardized mean difference (SMD) = 0.29), significantly elevated LYM count (SMD = 0.84) and D-dimer level (SMD = 0.35), as well as lower levels of fibrinogen (SMD = 0.42) and ALB (SMD = 0.27) compared to the internal validation set (all SMD > 0.1). These differences indicate that the external validation set presented a distinct baseline inflammatory and nutritional profile, which directly impacts the model’s transportability and contributes to the observed decline in discriminative performance.

### Model interpretation

Following systematic benchmarking of all candidate models, LightGBM was selected as the preferred algorithm for subsequent visual interpretation, aimed at disentangling feature-level drivers of the predictive decision process. The SHAP methodology was employed to estimate the marginal contribution of each predictor to the model output. The resulting importance matrix (**Figure 8A**) identified the six most influential features for anemia risk prediction—namely, LYM count, D-dimer, ALT, TBIL, sex, and ALB—in rank order. To clarify the valence of these effects, **Figure 8B** presents a SHAP summary plot, where each point represents an individual patient and color gradation from red to blue corresponds to descending feature values. The SHAP value along the abscissa exhibits a direct positive relationship with the estimated probability of anemia. In directional terms, higher D-dimer levels, female sex, outpatient admission, P, and fibrinogen were positively associated with anemia risk, whereas elevated LYM count, ALT, TBIL, ALB, NEUT count, MPV, UA, and older age were inversely associated. The translational utility of the model at the individual level is demonstrated in **Figures 8C, D**. In these panels, red coding signifies feature contributions that elevate anemia probability, while blue denotes contributions that lower it; f(x) corresponds to the aggregate SHAP score per case, and the base value represents the mean SHAP score over the entire cohort. A classification of anemia is rendered when f(x) surpasses the base value. As shown, **Figure 8C** correctly identifies a primary lung cancer patient receiving chemotherapy as having concurrent anemia, while **Figure 8D** accurately discriminates a non-anemic patient, thereby corroborating the model’s discriminative capacity at the single-patient level.

**Figure 8:**
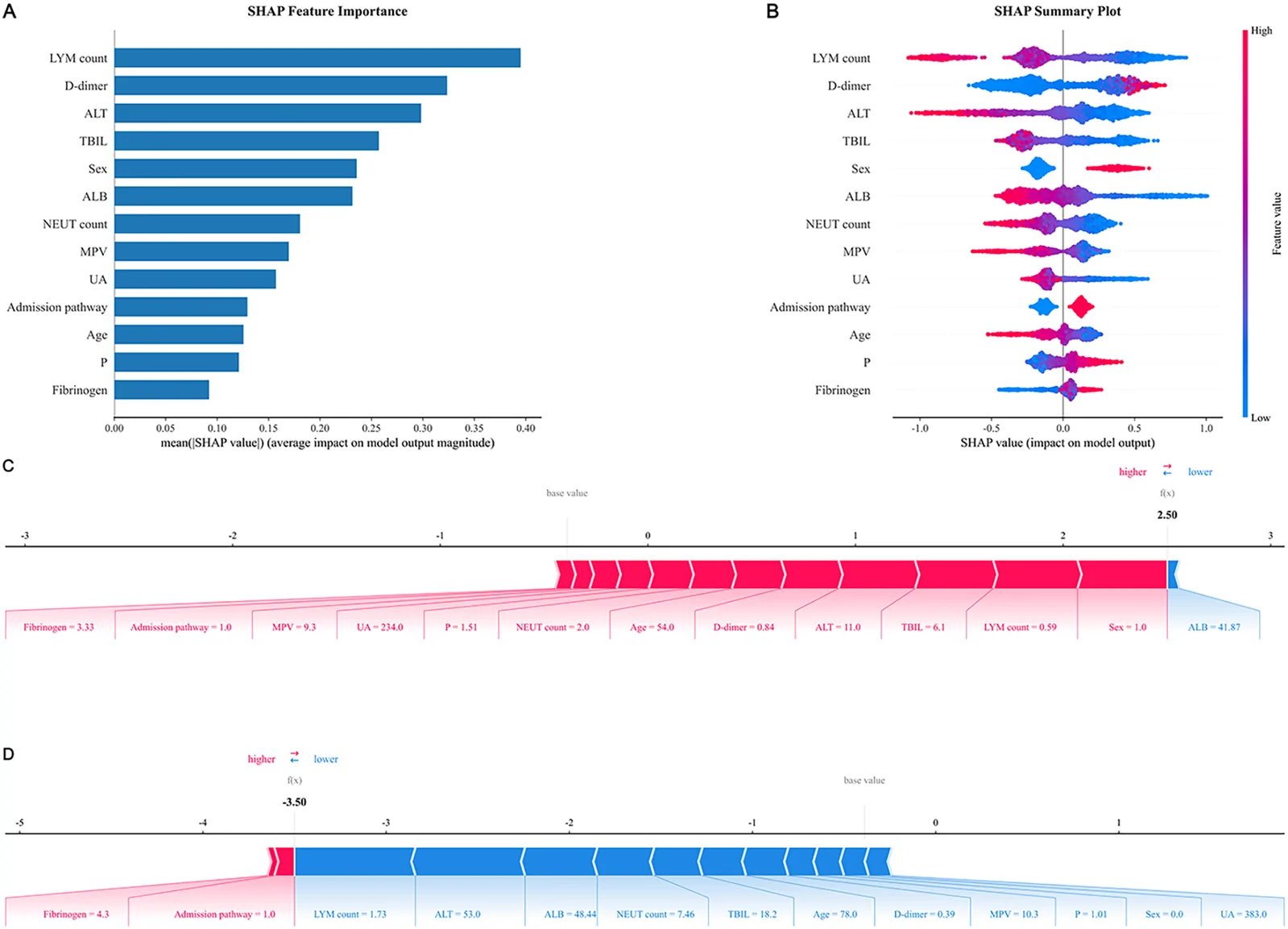
SHAP interpretation of the LightGBM model. **(A)** Feature importance ranking; **(B)** SHAP summary plot. Red and blue denote high and low feature values, respectively. SHAP values to the left of the vertical axis indicate reduced predicted risk of anemia, while values to the right indicate increased risk. SHAP force plot for individuals in the dataset at high **(C)** or low **(D)** risk of anemia.

## Discussion

In the present multicenter retrospective investigation, we developed and externally validated an ML-based prediction model for anemia risk in patients with primary lung cancer, integrating demographic, clinical, and laboratory features. Among nine competing algorithms, LightGBM was selected as the final model based on its comprehensive multi-dimensional performance. SHAP-based feature attribution further identified LYM count, D-dimer, ALT, TBIL, sex, and ALB as the predominant contributors, with distinct directional effects that align with pathophysiological plausibility. Collectively, these findings underscore the potential of the proposed model as a practical decision-support instrument for early risk stratification.

Among the nine competing ML algorithms, LightGBM consistently outperformed its counterparts across training, internal validation, and geographically distinct external validation sets, achieving AUCs of 0.871, 0.797, and 0.684, respectively. We acknowledge that in the internal validation set, several models, such as LR (AUC = 0.807), ANN (AUC = 0.806), and SVM (AUC = 0.802), achieved AUCs numerically comparable to or slightly higher than LightGBM (AUC = 0.797). The selection of LightGBM as the final model was therefore not solely based on its absolute AUC value in the internal set, but rather on its comprehensive multi-dimensional performance. Specifically, LightGBM demonstrated the most optimal balance among discrimination, calibration, and net clinical benefit. Furthermore, compared with black-box models like ANN or SVM, LightGBM offers practical advantages regarding computational efficiency and interpretability via SHAP analysis. Huang et al. employed RF, multilayer perceptron, and AdaBoost to predict chemotherapy-associated adverse effects in 403 NSCLC patients across 1,659 chemotherapy cycles, reporting AUCs of 0.75 for myelosuppression, 0.74 for hypoalbuminemia, and 0.76 for hepatic impairment, with the RF model demonstrating superior performance (8). Similarly, Sun et al. developed a risk prediction model for post-chemotherapy lung infection using multiple ML algorithms, finding that LR achieved an AUC of 0.888 (95% CI: 0.863–0.911) (11). More recently, Zhang et al. developed a CatBoost-based model for chemotherapy-induced neutropenia in NSCLC patients, attaining an AUC of 0.849 (95% CI: 0.788–0.905) (22). The superior performance of LightGBM in our study may be attributed to its gradient-based one-side sampling and exclusive feature bundling techniques, which enhance computational efficiency while preserving predictive accuracy, particularly in high-dimensional clinical datasets with complex nonlinear interactions (23). However, we must honestly acknowledge that an external AUC of 0.684 and sensitivity of 0.549 indicate modest rather than robust predictive performance when applied to a geographically heterogeneous cohort. This performance decay is primarily attributable to the significant baseline heterogeneity between the two centers (internal vs. external), particularly in sex distribution, LYM count, D-dimer, fibrinogen, and ALB (all SMD > 0.1), which is further amplified by the small proportion of external samples (9.8%). Despite this, an AUC of 0.684 remains clinically acceptable for a pre-treatment screening tool, as evidenced by our DCA, which shows a net clinical benefit within threshold probabilities of 0.15–0.50. Future prospective re-calibration and validation in diverse populations are mandatory before clinical deployment.

Compared with previous modeling approaches for chemotherapy-related toxicities, our study offers distinct advantages. Prior tools were often based on traditional logistic regression or lacked rigorous external validation. For instance, Vincent et al. developed a cycle-based anemia prediction tool for advanced NSCLC using logistic regression on 536 patients, relying primarily on pre-cycle hemoglobin and performance status without incorporating inflammatory or coagulation markers (24). Similarly, Gong et al. built a hematological toxicity model for lung adenocarcinoma using logistic regression, achieving high AUCs (0.904 and 0.902) but was restricted to a single treatment regimen and lacked external validation (25). More recently, ML models have been applied, yet they often focus on other toxicities (e.g., myelosuppression) or rely on single-center data (8, 22). By contrast, our study is specifically dedicated to anemia risk prediction in patients receiving platinum-based chemotherapy, incorporates a comprehensive panel of coagulation, inflammatory, and nutritional markers, and has been rigorously validated across two geographically distinct centers. The use of SHAP-based interpretability further enhances clinician trust by providing individual-level risk attribution, which is a critical step toward clinical adoption.

SHAP-based feature attribution showed that LYM count was the most strongly associated with anemia risk, followed by D-dimer, ALT, TBIL, sex, and ALB. The inverse association between LYM count and anemia risk is biologically plausible: lymphocytes play a central role in the hematopoietic niche, and chemotherapy-induced lymphopenia often parallels erythropoietic suppression through common progenitor cell damage (26, 27). Indeed, a prospective multicenter study by de Las Peñas et al. demonstrated that the GG genotype of HSPβ1 rs7459185 significantly predicted both acute lymphopenia ≥ Grade 3 (HR 1.590, 95% CI 1.004–2.517) and acute anemia ≥ Grade 2 (HR 1.886, 95% CI 1.060–3.356) in lung cancer patients receiving radiochemotherapy, suggesting shared genetic susceptibility pathways underlying these hematological adverse events (28). The positive association between D-dimer and anemia risk is likewise biologically coherent: platinum-based chemotherapy induces endothelial injury and activates coagulation cascades, with elevated D-dimer reflecting a prothrombotic and proinflammatory state that may exacerbate bone marrow microenvironment dysfunction (29). The inverse associations of ALB and liver function indices (ALT, TBIL) with anemia risk underscore the critical interplay between nutritional status, hepatic metabolic reserve, and hematopoietic capacity. Hypoalbuminemia, a surrogate for malnutrition and systemic inflammation, has been independently associated with adverse chemotherapy outcomes, while hepatic function impairment may compromise erythropoietin synthesis and bilirubin metabolism, further contributing to anemia development (30, 31).

Our SHAP-based directional analysis indicated that female sex, outpatient admission, elevated D-dimer, P, and fibrinogen were positively associated with anemia risk, whereas higher age, elevated LYM count, ALT, TBIL, ALB, NEUT count, MPV, and UA were inversely associated with anemia risk. The finding that female sex is associated with higher anemia risk is consistent with the well-documented sex dimorphism in chemotherapy toxicity profiles, potentially attributable to differences in body composition, drug metabolism, and baseline hemoglobin levels (32). Notably, the positive association between D-dimer and P with anemia risk, alongside fibrinogen, reinforces the concept that a procoagulant and inflammatory milieu predisposes patients to myelosuppression (33). The inverse association between age and anemia risk in our cohort, while seemingly counterintuitive, may reflect a “healthy survivor” effect, wherein older patients who tolerate chemotherapy may represent a selected subgroup with better baseline physiological reserve. Alternatively, younger patients often receive more intensive chemotherapy regimens, thereby incurring greater hematological toxicity (34). The negative associations of MPV, NEUT count, and UA with anemia risk are more nuanced: MPV, a marker of platelet activation, has been linked to systemic inflammation; its inverse association may reflect the complex interplay between megakaryocyte and erythroid lineages under treatment-related hematopoietic stress (35). Collectively, these directional findings provide hypotheses for future mechanistic studies and align with established pathophysiological frameworks.

The translational utility of the LightGBM model is further illustrated through its individual-level SHAP force plot analysis, which visually dissects the contribution of each predictor to a given patient’s predicted anemia probability. This capacity to provide case-specific, interpretable risk attribution addresses a critical gap in clinical decision support—moving beyond population-level risk estimates to patient-centered precision care (36). As Zhang et al. noted in their AI web application for neutropenia prediction, the integration of SHAP with model-agnostic explanation techniques substantially enhances clinician trust and adoption, as it enables users to understand not only the “what” but also the “why” of a prediction (22). Furthermore, the inclusion of an independent external validation set drawn from a geographically distinct institution is a methodological strength that distinguishes our study from most prior single-center ML models (37, 38). The modest but acceptable external AUC of 0.684 underscores the inevitable performance decay when models are deployed in heterogeneous real-world settings, yet it also affirms the model’s robustness beyond the derivation population. As systematically documented in a recent meta-analysis of clinical prediction model validation studies, models that perform well in internal testing frequently exhibit attenuated—yet often clinically meaningful—discriminative ability when applied to independent cohorts, underscoring the indispensable value of geographic and temporal validation prior to clinical deployment (39). Future work should focus on prospective deployment and seamless integration into EMR systems to facilitate real-time risk assessment and preemptive supportive care intervention.

Several limitations of this study warrant careful consideration. First, despite the inclusion of two geographically distinct institutions, the sample size of the external validation set (n = 337) was relatively modest, which may have constrained the statistical power to detect smaller effect sizes and could limit the generalizability of our findings to broader, more diverse populations. Second, the retrospective design inherent to EMR-based research introduces the potential for selection bias and information bias. Crucially, due to the inherent constraints of the retrospective electronic medical records, we were unable to accurately distinguish patients who developed incident anemia from those who had pre-existing anemia before treatment. This is a crucial limitation, as it means our model predicts overall anemia burden during early chemotherapy rather than strictly incident chemotherapy-induced anemia. We therefore reframe our model as a risk stratification tool for identifying high-risk patients prior to treatment initiation, rather than a predictive model for the causal toxicity of chemotherapy per se. Moreover, baseline hemoglobin levels were not consistently recorded in our structured EMR system across the participating centers and were therefore unavailable for inclusion as candidate predictors. Consequently, we could not exclude patients with baseline anemia or adjust for this strong predictor in our analyses, and thus a sensitivity analysis excluding patients with baseline anemia could not be conducted. The model captures the overall anemia burden in lung cancer patients and can effectively flag at-risk individuals for early intervention; however, future prospective studies with strictly longitudinal data are imperative to disentangle the causal relationship between chemotherapy and anemia. Consequently, unmeasured confounders—such as chemotherapy regimen specifics (e.g., drug types, dosages, cycle counts), cumulative drug exposure, and supportive care variations—were not accounted for in the current analytical framework. We intentionally excluded these dynamic, treatment-related variables from the feature set because the primary objective of this study was to build a practical, pre-treatment screening tool based solely on objective demographic and laboratory parameters routinely available at the time of admission. Specifically, we were unable to report the specific platinum-based regimens administered to patients (e.g., cisplatin vs. carboplatin, combination drugs, dosages, or number of cycles) due to the limitations of our structured EMR database. Given that these treatment-related factors are directly associated with the severity of hematological toxicity, their absence means our model reflects baseline host vulnerability (e.g., inflammatory and nutritional status) rather than direct chemotherapy toxicity. Therefore, the current model serves as a general screening tool to flag patients at risk of anemia prior to the initiation of any specific platinum-based regimen, rather than a tool to predict the exact hematological toxicity of a given treatment protocol. Future prospective iterations should incorporate these treatment-specific covariates to further refine and optimize predictive accuracy. Third, the laboratory measurements utilized in model development were obtained at a single time point (index admission) and may not fully capture dynamic fluctuations in biomarkers over the course of chemotherapy, potentially attenuating the model’s real-time predictive capacity in longitudinal clinical scenarios. Fourth, while the LightGBM model demonstrated satisfactory performance in the present cohorts, its applicability to other ethnic groups, healthcare systems, or treatment protocols (e.g., immunotherapy-combination regimens) remains untested. In addition, histological type (e.g., NSCLC vs. SCLC) was not available in our structured database and was therefore not included as a candidate predictor. Given the fundamental differences in biological behavior and treatment protocols between these subtypes, the potential influence of a heterogeneous study population on model performance cannot be entirely excluded. However, it should be noted that the 13 predictors retained by LASSO were predominantly objective laboratory parameters that reflect the patient’s physiological, nutritional, and inflammatory status rather than tumor histological characteristics. This suggests that the model is more likely to capture host vulnerability factors than subtype-specific classification signals. Nevertheless, future prospective studies should incorporate histological type and specific chemotherapy regimen details to further refine the model’s predictive specificity and applicability across different lung cancer subtypes. Future prospective studies with strictly longitudinal data and serial baseline hemoglobin measurements are imperative to disentangle the causal relationship between chemotherapy and anemia and to further validate the model’s performance specifically for incident events.

## Conclusions

In summary, this multicenter study provides evidence that a LightGBM-based prediction model, incorporating a comprehensive panel of readily accessible clinical and laboratory variables, can moderately and acceptably stratify the risk of anemia among patients with primary lung cancer prior to the initiation of platinum-based chemotherapy. The model demonstrates moderate, rather than robust, discriminative performance and satisfactory calibration across internal and external validation sets, and offers transparent interpretability through SHAP analysis. By enabling early identification of high-risk individuals, this tool holds potential promise for facilitating personalized supportive care strategies. However, given the modest external AUC of 0.684, cautious interpretation is required, and external validation in prospective, multi-ethnic, and real-world clinical settings is imperative before widespread clinical deployment can be recommended.

## Data Availability

The raw data supporting the conclusions of this article will be made available by the authors, without undue reservation.
