# Supplementary material for: Development and validation of a machine learning-based prediction model for anemia risk in patients with primary lung cancer receiving platinum-based chemotherapy: a multicenter study
Source: Front Oncol. 2026 Sep 30;16:1948751. doi: 10.3389/fonc.2026.1948751 (PMC13661483; doi:10.3389/fonc.2026.1948751)
Supplement: Supplementary file 1 [file Table1.docx]

**Table S1.** Missing rates of all candidate variables

| **Variables** | **Missing rate (%)** | **Handling** |
| --- | --- | --- |
| TC (mmol/L) | 37.84 | Excluded |
| TG (mmol/L) | 35.82 | Excluded |
| LDL-C (mmol/L) | 38.05 | Excluded |
| HDL-C (mmol/L) | 38.12 | Excluded |
| Myo (ng/mL) | 34.99 | Excluded |
| FBG (mmol/L) | 50.89 | Excluded |
| eGFR (mL/min/1.73m²) | 37.57 | Excluded |
| Cys-C (mg/L) | 42.37 | Excluded |
| GGT (U/L) | 35.57 | Excluded |
| TBA (µmol/L) | 42.14 | Excluded |
| Fibrinogen (g/L) | 13.92 | MICE imputation |
| D-dimer (mg/L) | 21.92 | MICE imputation |
| PLT count (×10⁹/L) | 0.74 | MICE imputation |
| MPV (fL) | 1.26 | MICE imputation |
| PDW (%) | 0.84 | MICE imputation |
| NEUT count (×10⁹/L) | 1 | MICE imputation |
| LYM count (×10⁹/L) | 0.71 | MICE imputation |
| MONO count (×10⁹/L) | 1.13 | MICE imputation |
| BUN (mmol/L) | 1.87 | MICE imputation |
| CREA (μmol/L) | 1.39 | MICE imputation |
| UA (μmol/L) | 1.42 | MICE imputation |
| ALT (U/L) | 1.78 | MICE imputation |
| AST (U/L) | 1.65 | MICE imputation |
| ALP (U/L) | 1.36 | MICE imputation |
| TBIL (μmol/L) | 1.68 | MICE imputation |
| ALB (g/L) | 1.32 | MICE imputation |
| GLB (g/L) | 1.32 | MICE imputation |
| K (mmol/L) | 1.68 | MICE imputation |
| P (mmol/L) | 11.53 | MICE imputation |
| Cl (mmol/L) | 1.68 | MICE imputation |
| Na (mmol/L) | 1.68 | MICE imputation |
| Ca (mmol/L) | 7.62 | MICE imputation |

Abbreviations: **TC**, total cholesterol; **TG**, triglycerides; **LDL-C**, low-density lipoprotein cholesterol; **HDL-C**, high-density lipoprotein cholesterol; **Myo**, myoglobin; **FBG**, fasting blood glucose; **eGFR**, estimated glomerular filtration rate; **Cys-C**, cystatin-C; **GGT**, gamma-glutamyl transferase; **TBA**, total bile acids; **PLT**, platelet; **MPV**, mean platelet volume; **PDW**, platelet distribution width; **NEUT**, neutrophil; **LYM**, lymphocyte; **MONO**, monocyte; **BUN**, blood urea nitrogen; **CREA**, creatinine; **UA**, uric acid; **ALT**, alanine aminotransferase; **AST**, aspartate aminotransferase; **ALP**, alkaline phosphatase; **TBIL**, total bilirubin; **ALB**, albumin; **GLB**, globulin; **K**, potassium; **P**, phosphorus; **Cl**, chloride; **Na**, sodium; **Ca**, calcium.

**Table S2.** Imputation results for variables retained in the training set (missing rate < 30%)

| **Variables** | **Pre‑imputation** | **Post‑imputation** | ***P* value** |
| --- | --- | --- | --- |
| Fibrinogen (g/L) | 3.93 (3.29, 4.84) | 3.9 (3.27, 4.8) | 0.457 |
| D-dimer (mg/L) | 0.45 (0.31, 0.8) | 0.44 (0.31, 0.8) | 0.756 |
| PLT count (×10⁹/L) | 213 (162, 272) | 213 (162, 272.5) | 0.993 |
| MPV (fL) | 9.2 (8.5, 10.1) | 9.2 (8.5, 10.1) | 0.944 |
| PDW (%) | 16.1 (15.7, 16.4) | 16.1 (15.7, 16.4) | 0.849 |
| NEUT count (×10⁹/L) | 3.46 (2.39, 4.74) | 3.47 (2.39, 4.72) | 0.997 |
| LYM count (×10⁹/L) | 1.06 (0.77, 1.42) | 1.06 (0.77, 1.42) | 0.973 |
| MONO count (×10⁹/L) | 0.42 (0.32, 0.55) | 0.42 (0.32, 0.55) | 0.927 |
| BUN (mmol/L) | 2.38 (1.96, 2.88) | 2.38 (1.96, 2.88) | 0.876 |
| CREA (μmol/L) | 62 (52, 73) | 62 (52, 73) | 0.961 |
| UA (μmol/L) | 324 (272, 385) | 324 (272, 385) | 0.934 |
| ALT (U/L) | 20 (14, 28) | 20 (14, 28) | 0.961 |
| AST (U/L) | 23 (18, 28) | 23 (18, 28) | 0.826 |
| ALP (U/L) | 81 (66, 97.5) | 81 (66, 97.5) | 0.990 |
| TBIL (μmol/L) | 9 (7, 11.9) | 9 (7, 11.9) | 0.997 |
| ALB (g/L) | 41.07 (38.71, 43.39) | 41.08 (38.71, 43.43) | 0.900 |
| GLB (g/L) | 27.27 (24.78, 30.09) | 27.3 (24.78, 30.09) | 0.960 |
| K (mmol/L) | 4.06 (3.82, 4.28) | 4.06 (3.82, 4.28) | 0.935 |
| P (mmol/L) | 1.15 (1.01, 1.29) | 1.14 (1.01, 1.28) | 0.374 |
| Cl (mmol/L) | 105 (103, 107) | 105 (103, 107) | 0.980 |
| Na (mmol/L) | 140 (138, 141) | 140 (138, 141) | 0.983 |
| Ca (mmol/L) | 2.26 (2.18, 2.34) | 2.26 (2.18, 2.34) | 0.938 |

Abbreviations: **PLT**, platelet; **MPV**, mean platelet volume; **PDW**, platelet distribution width; **NEUT**, neutrophil; **LYM**, lymphocyte; **MONO**, monocyte; **BUN**, blood urea nitrogen; **CREA**, creatinine; **UA**, uric acid; **ALT**, alanine aminotransferase; **AST**, aspartate aminotransferase; **ALP**, alkaline phosphatase; **TBIL**, total bilirubin; **ALB**, albumin; **GLB**, globulin; **K**, potassium; **P**, phosphorus; **Cl**, chloride; **Na**, sodium; **Ca**, calcium.

**Table S3.** Imputation results for variables retained in the internal validation set (missing rate < 30%)

| **Variables** | **Pre‑imputation** | **Post‑imputation** | ***P* value** |
| --- | --- | --- | --- |
| Fibrinogen (g/L) | 3.96 (3.29, 4.94) | 4.01 (3.3, 4.98) | 0.615 |
| D-dimer (mg/L) | 0.47 (0.32, 0.79) | 0.48 (0.32, 0.8) | 0.594 |
| PLT count (×10⁹/L) | 208 (155, 280) | 208 (155, 280) | 0.975 |
| MPV (fL) | 9.2 (8.5, 10) | 9.2 (8.5, 10) | 0.982 |
| PDW (%) | 16.1 (15.7, 16.4) | 16.1 (15.7, 16.4) | 0.979 |
| NEUT count (×10⁹/L) | 3.38 (2.35, 4.74) | 3.41 (2.35, 4.74) | 0.929 |
| LYM count (×10⁹/L) | 1.04 (0.75, 1.4) | 1.05 (0.75, 1.4) | 0.956 |
| MONO count (×10⁹/L) | 0.42 (0.31, 0.55) | 0.42 (0.31, 0.55) | 0.971 |
| BUN (mmol/L) | 2.38 (1.92, 2.88) | 2.38 (1.92, 2.88) | 0.930 |
| CREA (μmol/L) | 62 (52, 73) | 62 (53, 73) | 0.987 |
| UA (μmol/L) | 322 (266.25, 381) | 322 (266.25, 381) | 0.963 |
| ALT (U/L) | 19 (14, 27) | 19 (14, 27) | 0.907 |
| AST (U/L) | 22 (18, 28) | 22 (18, 28) | 0.999 |
| ALP (U/L) | 80 (65, 96) | 80 (65, 96) | 0.988 |
| TBIL (μmol/L) | 8.8 (6.95, 11.6) | 8.8 (7, 11.6) | 0.887 |
| ALB (g/L) | 40.89 (38.6, 43.08) | 40.85 (38.56, 43.08) | 0.870 |
| GLB (g/L) | 27.3 (24.9, 30.08) | 27.27 (24.87, 30.06) | 0.912 |
| K (mmol/L) | 4.04 (3.81, 4.28) | 4.04 (3.8, 4.28) | 0.953 |
| P (mmol/L) | 1.17 (1.02, 1.31) | 1.16 (1.01, 1.31) | 0.616 |
| Cl (mmol/L) | 105 (103, 107) | 105 (103, 107) | 0.987 |
| Na (mmol/L) | 139.85 (138, 141) | 139.8 (138, 141) | 0.918 |
| Ca (mmol/L) | 2.26 (2.18, 2.33) | 2.25 (2.17, 2.33) | 0.681 |

Abbreviations: **PLT**, platelet; **MPV**, mean platelet volume; **PDW**, platelet distribution width; **NEUT**, neutrophil; **LYM**, lymphocyte; **MONO**, monocyte; **BUN**, blood urea nitrogen; **CREA**, creatinine; **UA**, uric acid; **ALT**, alanine aminotransferase; **AST**, aspartate aminotransferase; **ALP**, alkaline phosphatase; **TBIL**, total bilirubin; **ALB**, albumin; **GLB**, globulin; **K**, potassium; **P**, phosphorus; **Cl**, chloride; **Na**, sodium; **Ca**, calcium.

**Table S4.** Variance inflation factor (VIF) and tolerance values for the initial set of candidate variables (pre-LASSO)

| **Variables** | **VIF** | **Tolerance** |
| --- | --- | --- |
| Age (years) | 1.388 | 0.72 |
| Sex | 2.318 | 0.431 |
| Admission pathway | 1.168 | 0.856 |
| Smoking history | 2.133 | 0.469 |
| Drinking history | 1.38 | 0.725 |
| Surgical history | 1.06 | 0.943 |
| DM | 1.111 | 0.9 |
| Hypertension | 1.172 | 0.853 |
| CHD | 1.119 | 0.894 |
| Chronic gastritis | 1.096 | 0.912 |
| Pneumonia | 1.135 | 0.881 |
| Fibrinogen (g/L) | 1.61 | 0.621 |
| D-dimer (mg/L) | 1.177 | 0.85 |
| PLT count (×10⁹/L) | 1.687 | 0.593 |
| MPV (fL) | 1.383 | 0.723 |
| PDW (%) | 1.156 | 0.865 |
| NEUT count (×10⁹/L) | 1.586 | 0.631 |
| LYM count (×10⁹/L) | 1.33 | 0.752 |
| MONO count (×10⁹/L) | 1.654 | 0.605 |
| BUN (mmol/L) | 1.266 | 0.79 |
| CREA (μmol/L) | 1.655 | 0.604 |
| UA (μmol/L) | 1.505 | 0.664 |
| ALT (U/L) | 2.131 | 0.469 |
| AST (U/L) | 2.119 | 0.472 |
| ALP (U/L) | 1.257 | 0.796 |
| TBIL (μmol/L) | 1.242 | 0.805 |
| ALB (g/L) | 1.793 | 0.558 |
| GLB (g/L) | 1.219 | 0.82 |
| K (mmol/L) | 1.296 | 0.772 |
| P (mmol/L) | 1.363 | 0.734 |
| Cl (mmol/L) | 3.346 | 0.299 |
| Na (mmol/L) | 3.443 | 0.29 |
| Ca (mmol/L) | 1.478 | 0.677 |

Abbreviations: **VIF**, variance inflation factor; **DM**, diabetes mellitus; **CHD**, coronary heart disease; **PLT**, platelet; **MPV**, mean platelet volume; **PDW**, platelet distribution width; **NEUT**, neutrophil; **LYM**, lymphocyte; **MONO**, monocyte; **BUN**, blood urea nitrogen; **CREA**, creatinine; **UA**, uric acid; **ALT**, alanine aminotransferase; **AST**, aspartate aminotransferase; **ALP**, alkaline phosphatase; **TBIL**, total bilirubin; **ALB**, albumin; **GLB**, globulin; **K**, potassium; **P**, phosphorus; **Cl**, chloride; **Na**, sodium; **Ca**, calcium.

**Table S5.** Performance metrics of nine ML models in the internal validation set

| **Model** | **AUC (95%CI)** | **Sensitivity** | **Specificity** | **Youden index** | **F1 Score** |
| --- | --- | --- | --- | --- | --- |
| LightGBM | 0.797 (0.763-0.831) | 0.798 | 0.667 | 0.465 | 0.613 |
| XGBoost | 0.794 (0.760-0.829) | 0.801 | 0.661 | 0.463 | 0.612 |
| GBDT | 0.799 (0.765-0.833) | 0.754 | 0.733 | 0.486 | 0.628 |
| RF | 0.777 (0.741-0.812) | 0.772 | 0.653 | 0.426 | 0.592 |
| LR | 0.807 (0.773-0.840) | 0.768 | 0.720 | 0.489 | 0.629 |
| DT | 0.690 (0.651-0.729) | 0.603 | 0.685 | 0.288 | 0.510 |
| KNN | 0.774 (0.738-0.810) | 0.695 | 0.716 | 0.411 | 0.583 |
| ANN | 0.806 (0.772-0.839) | 0.746 | 0.731 | 0.477 | 0.623 |
| SVM | 0.802 (0.768-0.836) | 0.824 | 0.650 | 0.474 | 0.617 |

Abbreviations: **AUC**, area under the ROC curve; **CI**, confidence interval; **LightGBM**, light gradient boosting machine; **XGBoost**, extreme Gradient Boosting; **GBDT**, gradient boosting decision tree; **RF**, random forest; **LR**, logistic regression; **DT**, decision tree; **KNN**, K‑nearest neighbors; **ANN**, artificial neural network; **SVM**, support vector machine.

**Table S6.** Performance metrics of nine ML models in the external validation set

| **Model** | **AUC (95%CI)** | **Sensitivity** | **Specificity** | **Youden index** | **F1 Score** |
| --- | --- | --- | --- | --- | --- |
| LightGBM | 0.684 (0.626-0.742) | 0.549 | 0.785 | 0.334 | 0.595 |
| XGBoost | 0.680 (0.621-0.739) | 0.500 | 0.805 | 0.305 | 0.566 |
| GBDT | 0.680 (0.621-0.739) | 0.423 | 0.836 | 0.258 | 0.513 |
| RF | 0.676 (0.618-0.735) | 0.528 | 0.779 | 0.308 | 0.577 |
| LR | 0.668 (0.609-0.728) | 0.493 | 0.785 | 0.278 | 0.551 |
| DT | 0.634 (0.574-0.699) | 0.423 | 0.728 | 0.151 | 0.471 |
| KNN | 0.613 (0.551-0.674) | 0.373 | 0.810 | 0.183 | 0.457 |
| ANN | 0.667 (0.608-0.726) | 0.528 | 0.790 | 0.318 | 0.581 |
| SVM | 0.672 (0.613-0.731) | 0.549 | 0.764 | 0.313 | 0.586 |

Abbreviations: **AUC**, area under the ROC curve; **CI**, confidence interval; **LightGBM**, light gradient boosting machine; **XGBoost**, extreme Gradient Boosting; **GBDT**, gradient boosting decision tree; **RF**, random forest; **LR**, logistic regression; **DT**, decision tree; **KNN**, K‑nearest neighbors; **ANN**, artificial neural network; **SVM**, support vector machine.

**Table S7.** Comparison of baseline characteristics between the internal validation set (n=930) and the external validation set (n=337).

| **Variables** | **Internal validation set (n=930)** | **External validation set (n=337)** | **SMD** |
| --- | --- | --- | --- |
| Age (years) | 63.18 ± 8.74 | 64.07 ± 9.30 | 0.1 |
| Sex (female), n (%) | 299 (32.15%) | 66 (19.58%) | 0.29 |
| Admission pathway (Outpatient), n (%) | 509 (54.73%) | 178 (52.82%) | 0.04 |
| Fibrinogen (g/L) | 4.21 ± 1.20 | 3.67 ± 1.34 | 0.42 |
| D-dimer (mg/L) | 0.82 ± 1.05 | 65.32 ± 163.23 | 0.35 |
| MPV (fL) | 9.34 ± 1.20 | 10.03 ± 1.03 | 0.62 |
| NEUT count (×10^9/L) | 3.81 ± 2.19 | 4.84 ± 2.85 | 0.4 |
| LYM count (×10^9/L) | 1.12 ± 0.50 | 1.65 ± 0.72 | 0.84 |
| UA (μmol/L) | 327.78 ± 89.16 | 281.01 ± 90.09 | 0.52 |
| ALT (U/L) | 23.37 ± 16.51 | 23.76 ± 24.82 | 0.02 |
| TBIL (μmol/L) | 9.78 ± 4.36 | 11.69 ± 6.22 | 0.35 |
| ALB (g/L) | 40.62 ± 3.79 | 39.34 ± 5.31 | 0.27 |
| P (mmol/L) | 1.16 ± 0.22 | 1.21 ± 0.21 | 0.22 |

Abbreviations: **SMD**, standardized mean difference; **MPV**, mean platelet volume; **NEUT**, neutrophil; **LYM**, lymphocyte; **UA**, uric acid; **ALT**, alanine aminotransferase; **TBIL**, total bilirubin; **ALB**, albumin; **P**, phosphorus.
